# A personalized Institutional Review Board Liaison Service: Evaluation over its initial 30 months

**Published:** 2020-06-29

**Authors:** Zainab Abedin, Alan Teller, Brenda Ruotolo, Kawthar Muhammad, Deborah F. Stiles, Rui Ferreira, Nancy Green

**Affiliations:** 1 Evaluation and Continuous Improvement Resource of the Irving Institute for Clinical and Translation Research, Columbia University, New York, USA; 2 Institutional Review Board of the Human Research Protection Office, Columbia University, New York, USA; 3 Office of Vice President for Research, Columbia University, New York, USA; 4 Regulatory Knowledge and Ethics Support (RKSER) of the Irving Institute for Clinical and Translation Research, Columbia University, New York, USA; 5 Department of Pediatrics, Columbia University, New York, USA

**Keywords:** Ethics, Institutional Review Board, research support, translational research

## Abstract

**Background:**

The aim of this study is to evaluate whether a dedicated Institutional Review Board (IRB) Liaison Service situated at our Institute’s central location could provide additional useful staff support to the investigator community for interactions with the IRB at various levels of protocol submission and review.

**Materials and Methods:**

Over a period of 2½ years, from January 2015 to June 2017, a total of 501 in-person consultations were performed during office hours, usually 25–30 per month. Most requests concerned new protocol development, IRB policy questions, and strategies for compliance or assistance in addressing IRB comments on returned protocols. We analyzed the results of a user evaluation survey for in-person consults and performed a focused in-depth analysis of the impact of the IRB Liaison Service.

**Results:**

Survey response rate was 43%. Results of 215 completed satisfaction surveys were 100% positive. Users were primarily study coordinators and investigators. Of a randomly selected sample of consultations analyzed in-depth for 67 unique protocols, 73% were subsequently approved within 14 days.

**Conclusion:**

National concerns about IRB-related research delays have led to the re-assessment of IRB review processes at institutional levels. Overall, we have found the Liaison Service to be a popular, useful addition to research support for a meaningful number of researchers, enhancing our already research-friendly environment. We plan to continue the service and the evaluation going forward. We will focus in the next phase on exploring whether the Liaison Service can reduce IRB approval times for protocols using its services and on providing support for the use of single IRBs for multi-site studies.

**The following core competencies are addressed in this article:**

Practice-based learning and improvement.

## INTRODUCTION

The Human Research Protection Office (“HRPO”) at our university’s academic medical center(the “University”) oversees the activities of the university’s seven Institutional Review Boards (collectively, the “IRB”). Here, we report on the evaluation of a novel resource developed to support researchers and facilitate efficient IRB review, the “IRB Liaison Service.” The aim of this study is to evaluate whether this dedicated IRB Liaison Service, situated at our Institute’s central location, could provide additional useful staff support to the investigator community for interactions with the IRB at various levels of protocol submission and review.

The IRB manages approximately 2200 new human research studies each year. At present, approximately 6300 studies have IRB approval or have been determined to be human subjects research that is exempt from federal regulatory requirements. A university-wide electronic research administration management system provides a web-based platform for IRB protocol submission, review, return and approval, and related correspondence and document management. This eSystem also provides the platform for oversight of other research-related administrative functions, for example, training, radiation safety, etc.^[1]^

The Clinical and Translational Science Award (“CTSA”) program is sponsored by the National Center for Advancing Translational Sciences (“NCATS”) of the National Institutes of Health.^[2]^ Columbia University’s CTSA, the Irving Institute for Clinical and Translational Research, is focused on “accelerating the translation of novel interventions” to improve the health impact of clinical and translational research.^[3]^ One major area of focus for the CTSA program is improving efficiencies in IRB review and oversight, and the Irving Institute has had a long-standing commitment to implementing IRB improvements. ^[4,5]^ Nationally, concern about IRB-related research delays expressed by NCATS, the CTSA institutions and others have led to an assessment of IRB review processes by individual institutions and multi-site consortia.^[6–11]^

To examine the university’s institutional experiences and challenges with respect to IRB review, we undertook an assessment several years ago of the reasons for delays in IRB protocol approvals and considered ideas for improving the IRB review process. At that time, we performed in-depth analyses, including interviews with key informants, of a number of protocols with prolonged approval times to understand how the time had been spent with HRPO/IRB staff, IRB members and researchers, the reasons for multiple protocol returns and perspectives from both faculty and staff involved with those protocols. Separately, research faculty were queried about their IRB experiences and the type of support that could be useful to them. Finally, descriptions of IRB support services at other institutions were collected at CTSA multi-site meetings.

Despite their heavy administrative load, the HRPO/IRB staff try to be responsive to inquiries from members of research teams about protocol submissions and returns. In addition, senior staff routinely provide scheduled educational sessions on topics such as “IRB 101,” “HIPAA requirements in Research,” “IRB protocol submission through Rascal,” and others.

We asked if the establishment of a dedicated IRB Liaison Service situated at the Irving Institute’s central location could provide useful support to the research community. IRB Liaison services could provide support for submission to and interactions with the IRB at various points during the protocol submission and review process, to supplement HRPO/IRB staff availability and other existing resources. Given the need for one-on-one support that was suggested through our prior analyses, the Irving Institute and the HRPO started the IRB Liaison Service.

### The Institutional Review Board liaison service

The IRB Liaison Service provides support to researchers preparing IRB protocols that are compliant with university policies and regulatory requirements. The service also supports researchers in responding to IRB review of protocols by explaining IRB comments and providing assistance in fashioning appropriate responses and/or implementing requested changes.

The goals of the IRB Liaison Service are to:

1. Provide personalized support to members of research teams in their interactions with the IRB, primarily during submission and in response to the return of protocols;
2. Assess whether the IRB Liaison Service could help reduce protocol approval times for those protocols receiving support. Given a large number of protocols handled by our IRB’s seven boards, in its initial phase, the Service was meant to gather data on review times but was not intended to reduce overall IRB protocol approval times.

The IRB Liaison Service became available for use by researchers in May 2015. The Irving Institute and HRPO each provided 50% of the salary of a full-time employee (the “Liaison”). As an HRPO/IRB staff member, the Liaison received thorough training on federal and state statutes and IRB policies and practices to prepare for the position. The Liaison had access to staff perspectives on review and policy issues and took part in HRPO/IRB staff meetings.

The IRB Liaison Service was geographically centrally located for the community of human subject researchers, near the CTSA offices and the CTSA’s clinical research resource at Columbia University Irving Medical Center (CUIMC). The IRB Liaison Service has focused on providing support to University faculty and clinical research coordinators. Students are able to attend consultations when accompanied by their principal investigator (PI) or faculty advisor or mentor.

The IRB Liaison Service was initially provided through in-person consultations at twice-weekly “walk-in” office hours and by appointment at any other time. In addition, support was provided through telephone or e-mail contacts. The use of the IRB Liaison Service nearly doubled month-to-month during the 1^st^ year. Consequently, beginning in January 2016, the Service was expanded to offer three weekly walk-in hours.

## MATERIALS AND METHODS

### User satisfaction surveys

During the development phase of the IRB Liaison Service, the Irving Institute’s Evaluation and Continuous Improvement Resource consulted with the Irving Institute’s Regulatory Knowledge and Ethics Support Resource and the HRPO to analyze the effectiveness of implementation and to identify possible roadblocks to service delivery. In June 2015, an online tracking and satisfaction survey was implemented for researchers to complete following a consultation. The goal was to gauge the use and usefulness of the Service, and to follow-up with researchers who might have additional questions. The brief survey asked questions about the role of users in study teams, the types of topics/questions posed to the Liaison, user satisfaction, the likelihood of future use and the identification of the means by which users had heard of the Service.

Users filled out the survey either in person at the end of the consultation, or by E-mail sent immediately following each in-person consultation. As some users had multiple consultations, including those before protocol submission, sorting consultations by IRB protocol number was not possible. For these reasons, multiple surveys may have been completed for the same protocol. The satisfaction survey was prepared in and analyzed using Qualities (www.Qualtrics.com).

### In-depth analysis

The impact of the IRB Liaison Service on IRB protocol approval times was analyzed for a random sample of protocols for which consultations were provided. All protocols, with an associated protocol number that had one or more in-person IRB Liaison Service consultations from May 2015 to June 2017, were assigned a number in an Excel file. Using a randomization formula, a subset of 90 protocols were selected among consultations for further analysis with our intent being to maximize the number of protocols analyzed without impeding on consultation time. Protocols that did not result in an IRB submission and duplicate entries were excluded, resulting in a final dataset of 67 protocols. Those protocols were assessed by review process (expedited or full board review), by status (new submission, first return, second or subsequent return) and by which of the seven IRB committees was responsible for the review process.

**Figure 1:**
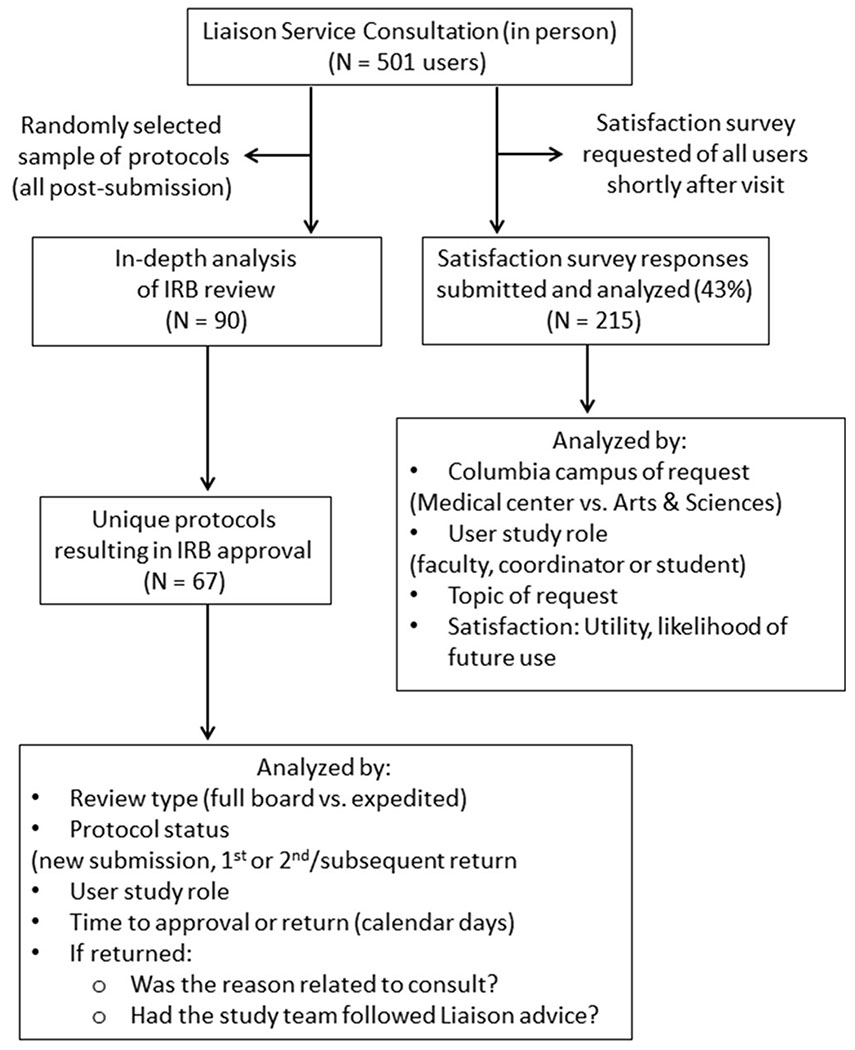
Study procedures for Institutional Review Board Liaison Service evaluation (January 2015–June 2017)

The Liaison drafted a summary of each meeting to describe the consultation that was conducted during the office hour sessions. Each protocol was reviewed using the Liaison’s notes to capture the issues raised in the meeting with users. These notes were compared to the IRB correspondence sent to researchers about issues raised from the review of the subsequent submission. The authors determined that this study did not require IRB/Ethics Committee Review. Figure 1 illustrates the study procedures for the evaluation of the Service.

## RESULTS

### Use of the Institutional Review Board liaison service

Generally, two consultations were provided per office hour session, approximately 25–30 consultations/month. Of the 215 users responding to the user surveys, 45% were research coordinators, 38% were faculty, and 16% were students. All those seeking consultations were from CUIMC and not from the arts and sciences campus.

### User survey

**Figure 2:**
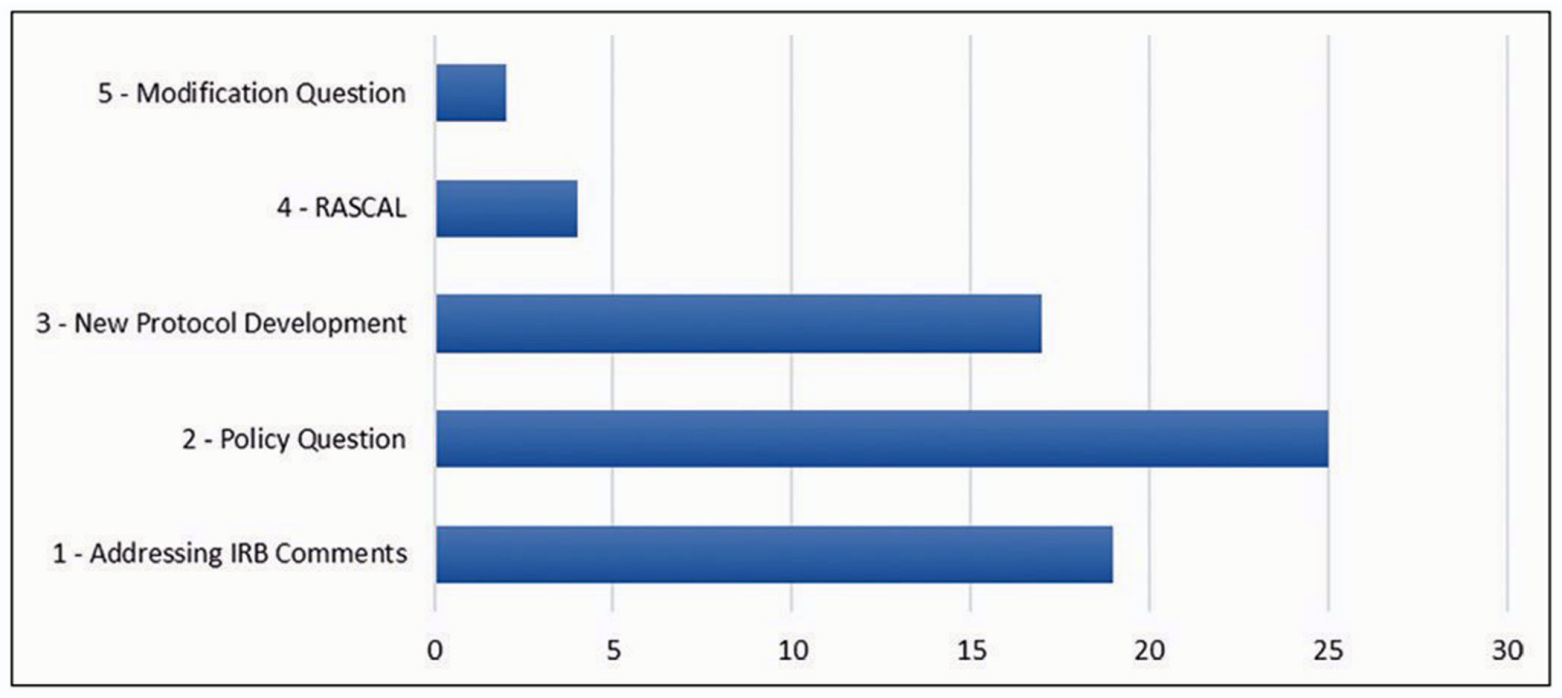
For the user survey, the proportion of consultations, by topic, is depicted here. The majority of queries pertained to policies on human subject research, development of new protocols and addressing Institutional Review Board comments. Rascal is Columbia’s web-based platform for all Institutional Review Board transactions, including submission, review, return and approval

The majority of user queries pertained to policies on human subjects research, development of new protocols and IRB comments on returned protocols [Figure 2]. Topics discussed during consultations covered all of the phases of IRB review. Frequently posed questions were:

- What criteria will the IRB use for reviewing my protocol?
- What are the requirements of the university’s data security policy, and how can I implement them for my research?
- Based on university, state, and federal IRB policies, what research procedures are needed to address the handling of incidental findings, genetic research, research with minors and other vulnerable populations?
- What are acceptable and appropriate recruitment methods?
- Where can I find the recommended language for use in consent documents?

**Figure 3:**
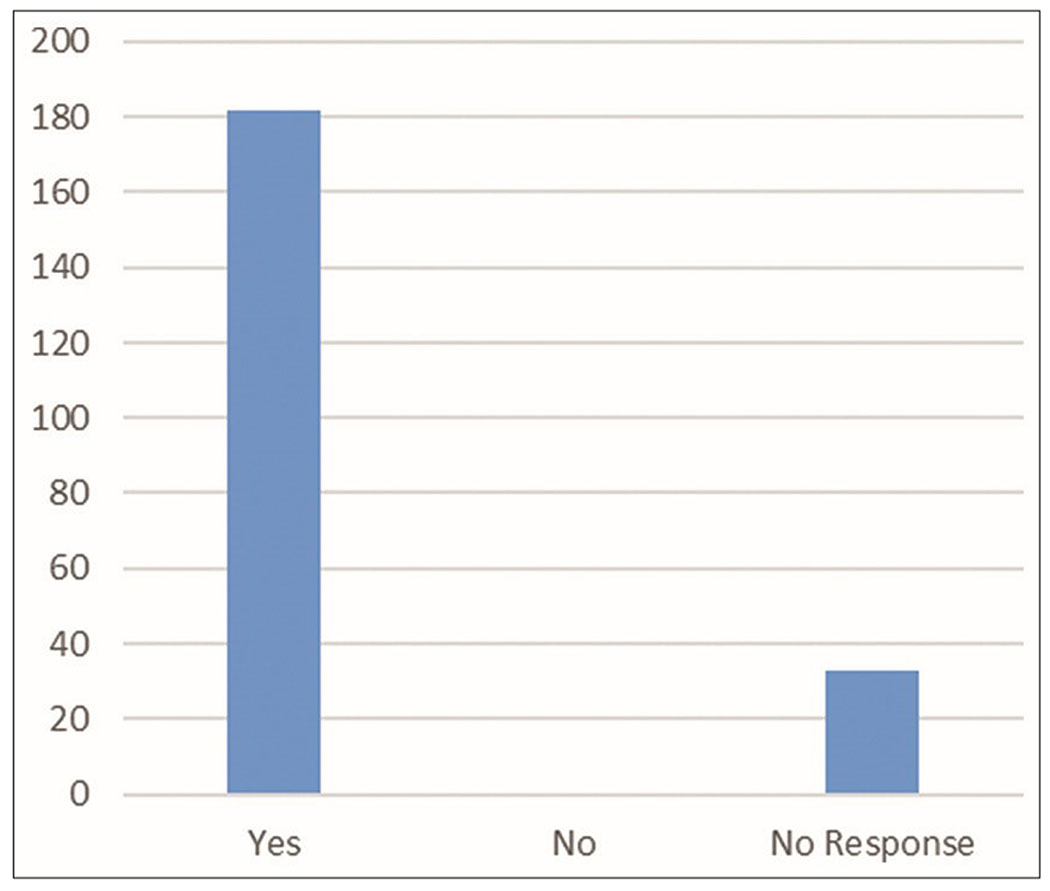
Liaison service user satisfaction, when asked: “did you find the service useful?” (*n* = 215). Proportion of responses: 85% positive, none negative. Data are virtually identical to responses when asked about the likelihood of returning for future Institutional Review Board-related consultations (Yes or likely, No or unlikely, No response)

Since the implementation of the IRB Liaison Service in May 2015, the response from users about their experience with the Service has been overwhelmingly positive [Figure 3]. The vast majority of users indicated that they were likely to return for future IRB-related consultations [data not shown, but the data were similar to those depicted in Figure 2].

An on-going issue has been ensuring that researchers are aware of the existence of the IRB Liaison Service. This is especially challenging at the university and other large academic institutions. The most common sources of knowledge about the service were e-mail announcements, followed by word-of-mouth and prior consultations. Periodic outreach efforts through broadcast e-mails, flyers, presentations, and website postings were generally met with a flurry of requests for the use of the service.

### In-depth analysis

The impact of the Service was assessed for its effect on IRB protocol approval or return and the time to protocol approvals. A detailed analysis was performed of each of the 67 randomly selected consultations, all of which were protocols that had already been submitted for review. For each protocol, we recorded who participated in the consultation [Table 1], the number with returns postconsultation [Table 2], the reasons for return(s), and the number of days from consultation to approval [Table 3].

In this sample, half of the users were coordinators and another one-third were faculty investigators, some as PIs [Table 1]. More than half of the protocols submitted to our eSystem postconsultation were for initial protocol approval [Table 2].

Following the consultation, 37% of submitted protocols were returned by the IRB. Of those returned, the majority (68%) were new protocols [Table 2]. Most of these protocols were returned because of issues other than those raised in the consultation. Interestingly, the same topic was referred to in 8 (32%) of the returned protocols. Reasons for consultation on the same topic varied. Some investigative teams had not followed the advice given. Others teams submitted responses to the IRB that did not clearly or adequately address the issue(s) that had been raised by reviewers.

Half of the 67 protocols in this sample received IRB approval within 9 calendar days following submission, and a total of three-quarters within 19 days [Table 3]. All of the remaining protocols were approved; however, for some, approval was obtained after a more than 1-month delay.

Finally, we attempted to address whether the IRB Liaison Service shifted the types of questions addressed to HRPO/IRB staff members. However, comparison data were not available, as routine tracking of the number or types of questions received by staff was not performed.

## DISCUSSION

Based on an assessment of the demand of investigators performing human subjects research for assistance in preparing and reviewing protocols, our IRB Liaison Service was created as a personalized on-site link between the IRB and the university researchers to address these needs. Analysis of the user surveys and for a randomly selected sub-set, the outcomes of the use of the Service since its launch in 2015 provided data for this evaluation.

Overall, the IRB Liaison Service received resoundingly positive reviews in the user surveys. Our in-depth analysis strongly suggests that the majority of the issues in returned protocols addressed in consultations were found to be acceptable by HRPO/IRB staff in subsequent submission. Postconsultation returns may be results of the same issues addressed by the Service or one or more additional issues raised in IRB review.

For the in-depth analysis, we considered comparing approvals of protocols that had been assisted by the service to those not assisted by the service. We decided not to follow through with this comparison due to several factors: the variety and type of IRB protocols; the feasibility of performing an in-depth review of protocols with appropriate matching of all variables; the existence of additional protocol issues beyond those that were addressed by use of the Service; and in unusual cases, review by a different IRB.

### Additional considerations

#### Reduced time for protocol preparation

The impact of the IRB Liaison Service on protocol approval times is only part of the impact assessment that is needed for human subject research reviews. Protocol development and submission and responses to IRB reviews require complex tasks. For example, considerable time is needed to provide support for junior researchers in investigator-initiated studies to develop IRB-compliant and complete protocols, including the informed consent forms, surveys and study recruitment materials, and HIPAA authorization forms, in ways that are consistent with the literacy level of the target subjects. Because of these issues, some less experienced researchers may be particularly discouraged in their human subjects research. To help address the need for the development of compliant consent forms, in addition to the liaison service, the university’s IRB has expanded the repertoire of on-line templates available for use.

The introduction of the IRB Liaison Service did not appreciably reduce the number of queries to HRPO/IRB staff regarding policies or specific protocols, nor was there a marked reduction in attendance at scheduled IRB education sessions. These findings support the conclusion that training and additional support beyond the service is still needed.

### Limitations

The user satisfaction survey may be biased in favor of those with favorable evaluations. However, the large proportion of the voluntary surveys submitted relative to the number of IRB Liaison Service users suggests that the results were subject to minimal bias. Additional consultations by the liaison were performed by telephone and e-mail, but evaluation was not performed due to a predicted low rate of returning the survey. The pool of researchers using the Service may exclude those with different perspectives on the value of the support that the Service was meant to provide.

The in-depth analysis was limited by the following features: the modest number of protocols analyzed; the multiple issues that may have arisen in protocol review; the selection of issue(s) that were selected by the researcher seeking liaison support; the type and scope of the research; the level of experience of the research team; and at what point in the IRB approval process the consultation was requested (e.g., during the first or second return). These and additional issues mentioned above precluded a parallel analysis of a bona fide comparison group.

## CONCLUSION

Overall, the IRB Liaison Service has become a critical addition to the support that we provide for human subjects research through the Irving Institute – HRPO/IRB collaboration. Awareness of the availability of the Service still needs to be improved through outreach to the university’s research community, particularly in light of the constant arrival of new researchers and coordinators. We plan to continue both the service and its evaluation to monitor its use and progress in improving the submission of high-quality protocols for human subjects research to the IRB.

As with most other research services provided by the Irving Institute and the HRPO, the IRB Liaison Services are provided reactively, i.e., by request. The next phase of the liaison service implementation will be to select specific strategies to more deliberately address the impact of the Liaison Service on IRB approval times for certain types of protocols. Priorities will be guided by input from regular detailed information about types of protocols that have excessive approval times. On-going input will be provided by leadership, including some of the co-authors of this paper, from the Irving Institute and the HRPO, as well as from the University research administration. For example, we are examining the need to provide support to investigator teams for submission of protocols utilizing single IRBs (sIRBs) for multi-site clinical trials, both in the form of increased use of templates and by targeted consultations. Our aspirational goal is for the Service to reduce times for IRB approval through investigator preparation and submission of high-quality protocols.

To further enhance the impact of the IRB Liaison Service, we will employ three approaches: (1) continuation of pro-active guidance to researchers in areas of high priority, for example, sIRBs; (2) continuation of the by-request service, based on results of the user satisfaction surveys; and (3) to increase the targeted capacity of the liaison service, to determine which services are better supported by departmental resources, rather than by the Service. An example is the guidance that is often needed by junior researchers as to how to use the university’s web-based research administration and compliance eSystem for HRPO/IRB transactions.

In summary, initiation and evaluation of the Irving institute-HRPO IRB Liaison Service have been demonstrated to result in high user satisfaction. Version 2.0 of the IRB Liaison Service will incorporate the results of our evaluations as well as existing research services provided by the Irving Institute to more strategically enhance its impact on our research.

## Figures and Tables

**Table 1:** From the in-depth analysis, liaison consultations by user role

User role	Number of protocols (%)	Number returned*
Coordinator	33 (49)	11
PI	14 (21)	4
Investigator	8 (12)	4
Student	12 (18)	6
Total	67	25

Half of the users were coordinators.

* Protocols returned after a consultation.

PI = Principal investigator

**Table 2:** From the in-depth analysis, the numbers each of protocols and returns, by the type of submission at initial consultation

Submission type	Number of protocols (%)	Number returned*
New protocol	38 (57)	17
Modification	20 (30)	5
Renewal	7 (10)	2
Closure	2 (3)	1
Total	67	25

Most of the returns were new protocols.

* Protocols returned after a consultation

**Table 3:** From the in-depth analysis, the number of days from consultation to institutional review board approval, 73% by 19 days

Days from consult to approval or determination	Number of protocols (%)
0-9	33 (49)
10-19	16 (24)
20-29	6 (9)
30-39	4 (6)
≥40	12 (18)
Total	67

Of the total, 25 (37%) were returned after consultation
